# The blind men and the elephant: recognising the multisystem symptoms of myotonic dystrophy type 1

**DOI:** 10.1186/s13023-025-03920-z

**Published:** 2025-08-13

**Authors:** Kristofoor E. Leeuwenberg, Johanna E. Bruijnes, llse Karnebeek, Fran Smulders, Sandra Altena-Rensen, Caroline M.L. Gorissen-Brouwers, Sylvia Klinkenberg, Catharina G. Faber, Hilde Braakman, Karlien Mul

**Affiliations:** 1 Department of Neurology, Donders Institute for Brain, Cognition and Behavior, Radboud Research Institute for Medical Innovation, Nijmegen, The Netherlands; 2 Department of Neurology, School of Mental Health and Neuroscience, Maastricht University Medical Center+, Maastricht, The Netherlands; 3 Department of Pediatric Neurology, Amalia Children’s Hospital, Radboud University Medical Center & Donders Institute for Brain, Cognition and Behavior, Nijmegen, Netherlands

**Keywords:** Myotonic dystrophy, Clinical, Diagnosis

## Abstract

Although myotonic dystrophy type 1 (DM1) is named after its characteristic muscle symptoms, it is in fact a multisystem disorder that can affect many different organs. It is therefore not surprising that this disease can manifest with a myriad of symptoms, depending on the organs involved. The age of onset and severity of symptoms vary widely. Diagnostic delays of more than ten years are common and it’s not unusual for an entire family to be diagnosed only after the birth of a child with a severe phenotype. Knowledge of the spectrum of possible symptoms in DM1 can aid clinicians to recognise this disorder, thereby preventing unnecessary diagnostic delay and facilitating early treatment of disease complications. Here, we present an overview of the potential symptoms of DM1 at different ages, with the aim of raising awareness among healthcare professionals about the recognition of this disabling disease.

## Introduction

Myotonic dystrophy type 1 (DM1, also known as Steinert’s disease) is the most common form of inherited muscular dystrophy in adults [1]. The disease comprises a broad clinical spectrum regarding both the severity and the type of symptoms.

Differences in disease severity are, at least in part, because of the instability of the autosomal dominantly inherited expansion of a CTG-repeat in the *myotonic dystrophy protein kinase (DMPK)* gene [2]. This instability often leads to an increase in the repeat expansion when it is passed on to the next generation resulting in an earlier disease onset in subsequent generations. This phenomenon is called genetic anticipation.

Differences in the type of symptoms are related to the multiorgan involvement in DM1. Many organs in the body can be affected, including skeletal as well as smooth muscle, the heart and respiratory system, the endocrine system and the central nervous system. Because of the multisystem involvement, patients first present at many different medical disciplines before a physician may realise that all the separate medical problems are the results of one overarching cause, namely DM1. Varying presentations by age of onset prevent a uniform diagnostic approach. Late-onset patients with mild symptoms can be difficult to recognise and may not be diagnosed until a child with a more severe phenotype is born into the family. On the other hand, the predominant symptoms in babies and children may be so different from adults, that recognising the disease in children may pose a diagnostic challenge as well. Regional differences in healthcare and disease knowledge may contribute to delays as well. Taking all of this into account, it is not surprising that diagnostic delays of many years are common (Box 1) [3, 4]. 

Although there is currently no cure for DM1, complications such as cardiac conduction defects can be treated effectively, with consensus-based care recommendations available [5, 6]. An early diagnosis can also help to provide affected people with adequate information and counselling about their risks of having a child with more severe DM1. Therefore a delay in diagnosis can have serious consequences for the patient. This article aims to assist clinicians in recognising DM1 by discussing the entire disease spectrum and highlighting key examination clues. Improved recognition by physicians can reduce diagnostic delays, addressing both disabling and life-threatening complications earlier.

**Box 1:** Clinical cases with delayed diagnosis of DM1. These cases are inspired by real clinical experiences, but details have been altered to prevent possible identification

Case 1	A 33-year-old woman, who visited an ophthalmologist for visual complaints because of cataracts three years earlier, gives birth to her son. Immediately after birth, the child experiences respiratory problems, along with hypotonia and feeding difficulties. This prompts extensive diagnostic testing, leading to the diagnosis of myotonic dystrophy type 1 first in her son and subsequently in herself. Diagnostic delay = 3 years.
Case 2	A 38-year-old female has a documented family history of myotonic dystrophy type 1. From the age of 28 she was consecutively diagnosed with irritable bowel syndrome, fatigue, and excessive sleepiness, for which a wide range of diagnostic tests have already been conducted. After 10 years, the patient returns at the suggestion of a family member to inquire whether her symptoms might be related to myotonic dystrophy, and the diagnosis is ultimately confirmed. Diagnostic delay = 10 years.
Case 3	A 72-year-old man has a medical history that includes a pacemaker, obstructive sleep apnoea (OSA), dysarthria attributed to a previous stroke, and distal muscle weakness diagnosed as polyneuropathy. The onset of symptoms was around retirement at the age of 65. He now presents with concerns about nocturnal breathing difficulties and casually notes that he struggles to open his hands after shaking hands. This eventually leads to an overarching diagnosis of myotonic dystrophy type 1 explaining his medical history. Diagnostic delay = 7 years.
Case 4	An 11-year-old boy has a medical history that includes a global developmental delay, learning difficulties, behavioural problems, urinary incontinence and constipation. Neurological examination revealed facial weakness, dysphagia, dysarthria, weakness of his neck flexors, hands, and feet dorsiflexors. The symptoms that had been present since his first year of life turned out to be attributed to myotonic dystrophy type 1. Diagnostic delay = 10 years.

**Table 1:** Multiorgan involvement in DM1

Organ system	Main clinical features
Central nervous system	Fatigue Excessive daytime sleepiness Chronic pain Sleep disorders Mood disorders Cognitive impairment Speech & language impairment Apathy Reduced disease awareness Behavioural problems (ADHD, anxiety, autism spectrum disorders or other social communication disabilities)
Ocular	Cataracts
Face	Bilateral ptosis Atrophy of temporal muscles Frontal balding (men) Reduced facial expressions Oral health issues Dental misalignment / orthodontic issues
Oropharyngeal	Dysarthria Dysphagia Hypersalivation High palate
Musculoskeletal	Myotonia Myalgia Muscle cramps Muscle weakness Scoliosis
Cardiac	Conduction disorders Atrial or ventricular arrhythmias Cardiomyopathy
Respiratory	Dyspnoea Sleep-related breathing disorders (sleep apnoea and nocturnal hypoventilation) Weakened cough strength Pneumonias Diaphragm weakness and respiratory failure
Gastrointestinal	Abdominal pain Vomiting Obstipation / pseudo-obstruction Diarrhoea Faecal incontinence Bloating and flatulence Cholelithiasis Mildly elevated liver enzymes
Urological	Urinary incontinence
Reproductive	Fertility issues Menstrual disorders Pregnancy and childbirth complications
Endocrine	Diabetes mellitus Thyroid disorders Hyperlipidaemia Reproductive hormonal dysfunction
Other	Increased risk of malignancy Pilomatrixoma Anaesthesia complications

### Clinical features of DM1

Generally, DM1 is a slowly progressive disease. Symptoms can manifest at any age, although the most common presenting symptoms differ depending on the age at onset. Based on the age at onset DM1 patients are stratified into four different subtypes [7]: congenital, childhood, adult-onset and late-onset. Previously, a distinction was occasionally made between infantile (1 month − 10 years) and juvenile (10– 18 years) in the childhood subtype. Each subtype is discussed separately below. What all patients with DM1 bear in common is the multiorgan involvement (Table 1), with each system potentially showing symptoms that offer diagnostic clues. Life expectancy is reduced compared to the general population with a mean age at death in the mid-fifties, though again highly variable depending on the disease severity [8]. 

Below, some of the main symptoms for each disease subtype are discussed. It is important to realise that due to the broad spectrum of symptoms, many patients with DM1 will initially seek care from medical specialties other than neurology. While the symptoms seen in DM1 patients are not necessarily common, their complaints are often misattributed to other conditions that are widespread in the general population such as irritable bowel syndrome, fibromyalgia, age-related cataracts, or stress-related sleep and school difficulties. This can be misleading, and it is therefore of utmost importance to always critically review a patient’s medical history. Commonly, the main clue to the diagnosis of DM1 lies in the combination of multiple previous medical problems, such as obstructive sleep apnoea, cataracts, cardiac conduction disorders for which a pacemaker may be indicated, fatigue, irritable bowel syndrome, and many more. In itself, many of these problems would not routinely trigger the work-up for a neuromuscular disease. On the other hand, many symptoms of DM1 are not routinely asked for in patients that first present with the suspicion of a muscle disease. The family history can reveal important clues, but only when the appropriate questions are being asked, including not only muscular features, but also for example early-onset cataracts or sudden unexplained or cardiac death. Gathering a medical history in patients with DM1 can be complex, as individuals with the condition may have reduced awareness about their symptoms, possibly influenced by cognitive factors, which can result in underreporting of symptoms [9]. Taking all of this into account, it is important for physicians to recognise clues that can hint towards DM1. In this way, the history and physical examination can be focused on searching for additional signs of DM1.

### Adult-onset DM1

In the adult-onset subtype the first symptoms of DM1 appear in the second to fourth decade of life [7]. The most common presenting symptom is myotonia (54%), followed by muscle weakness (21%), fatigue (11%) and cataracts (4%).^10^ From the viewpoint of patients, problems with mobility and daytime alertness may be experienced as major burdens [11]. 

#### Muscular involvement

The pattern of muscle disease is distinct from many other muscle diseases, and is characterised by predominantly distal weakness. The neck flexor muscles are affected early as well.

**Fig. 1:**
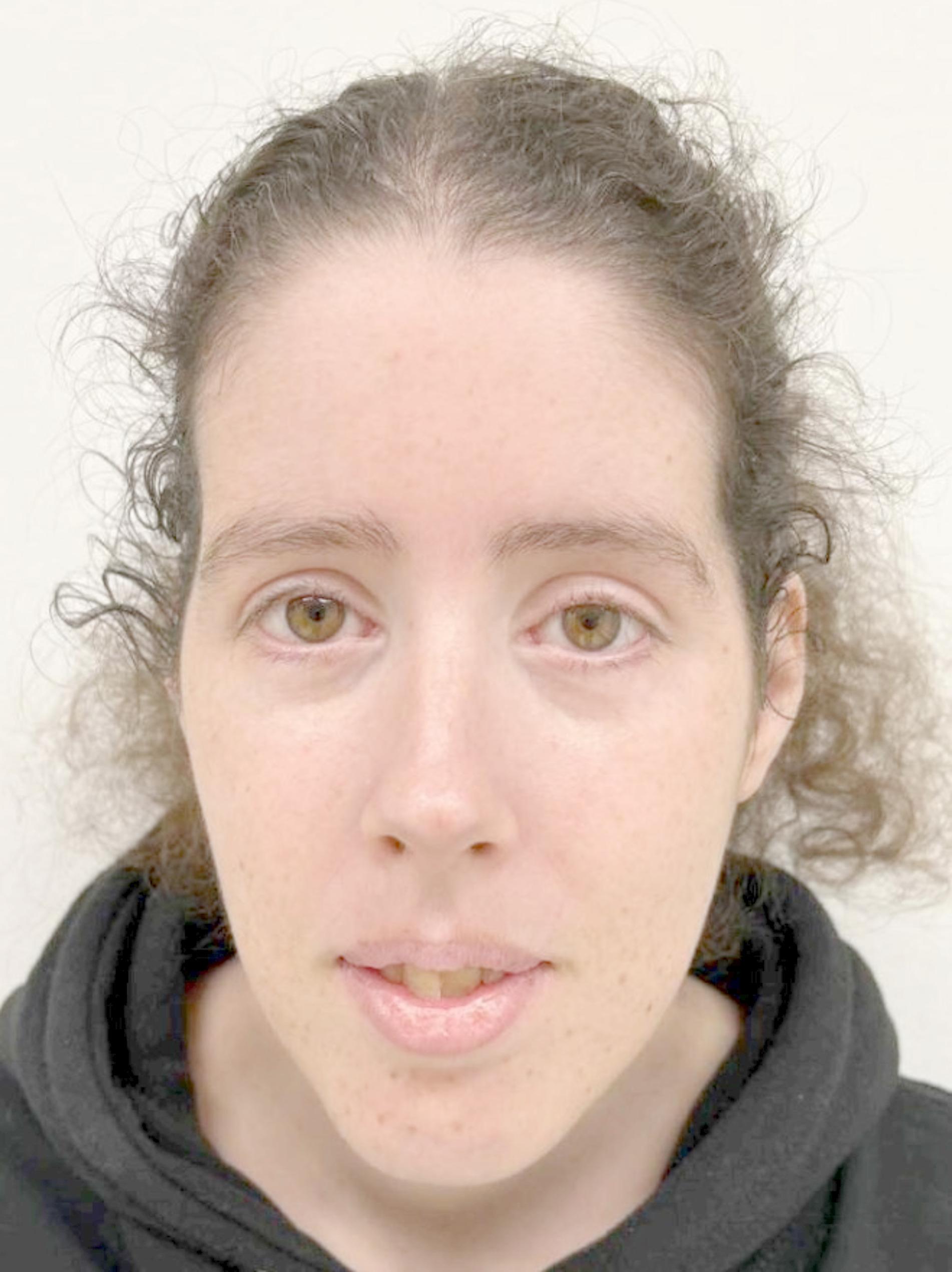
Photograph of a 33-year-old woman with DM1, with characteristic facial features such as temporal atrophy, a long and thin facial contour, and ptosis of her left eye

Facial weakness, along with other characteristic features of DM1, can contribute to an appearance that may seem tired or expressionless. The facial signs vary from subtle, sometimes only acknowledged as sign of the disease *after* a patient is diagnosed, to severe myopathic facies. Typical features include bilateral ptosis, difficulty closing the eyes completely, and frontal balding in men. Temporal wasting gives rise to a long, thin facial contour (Fig. 1) [12]. 

Myotonia is another significant characteristic of muscle involvement in DM1, and a common presenting symptom of the disease. Patients may present with complaints of difficulty releasing grip on objects, opening their eyes, beginning to speak or ‘jaw locking’ [2]. Many report a sense of stiffness in the muscles, especially in the hands. The myotonia is often worsened by cold and improves with repeated muscle contraction, which is referred to as the ‘warm-up phenomenon’ [13]. Clinicians should be aware that patients can experience myotonia yet may not actively report it. On physical examination myotonia can show as grip or eyelid myotonia: a delayed relaxation after tightly squeezing either the flexor muscles of the hand or the sphincter muscles around the eyes. Myotonia can also be found through percussion of the thenar eminence, forearm extensors, or tongue. Sometimes it is also noticed as having trouble to initiate speaking.

Patients with DM1 often have a dysarthric speech and a nasal tone of voice, because of weakness of the pharyngeal muscles [14]. Especially dysarthria is commonly attributed to other diagnoses, for example the suspicion of a (minor) stroke in the elderly patient. The pharyngeal muscle weakness gives rise to dysphagia. Patients tend to compensate by adapting their eating habits and might only report trouble swallowing when it is severe.

#### Cataracts

Another clinical hallmark of DM1 is cataracts. Cataracts tend to be one of the initial symptoms of the disease and as such, patients may first present at an ophthalmologist [4]. Patients describe symptoms of cataracts as blurry or cloudy vision and glare around lights. Especially when the onset is below 55 years of age, there is a positive family history for early cataracts, and on examination it has the typical ‘Christmas tree appearance’ featuring punctate iridescent opacities in the lens, this should raise a suspicion of underlying DM1 [15]. More advanced cataracts in DM1 may resemble senile cataracts, and can sometimes be difficult to differentiate.

#### Fatigue and sleep

The symptom of DM1 that is consistently being reported by patients as one of the most burdensome is fatigue [11, 16]. Up to 90% of patients with DM1 experience fatigue. The cause is multifactorial and may be associated with muscle weakness and excessive fatigability after physical exertion, as well as respiratory failure, sleep disturbances and mood disorders. Fatigue can also be a presenting symptom, preceding muscle weakness [17]. Patients often experience both fatigue (a lack of physical energy) and sleepiness (an inability to remain awake), which should be considered separate symptoms. Excessive daytime sleepiness can cause significant morbidity with patients falling asleep for example during work or social activities. Similar to fatigue, sleepiness is multifactorial in nature and DM1 is associated with multiple sleep disorders, including central and obstructive sleep apnoea and hypoventilation, poor sleep hygiene and deprived sleep quality [18]. 

#### Respiratory involvement

About one-third of adult-onset patients have respiratory involvement marked by reduced pulmonary function [10]. However, the overall prevalence of respiratory involvement is a lot higher when sleep-related breathing disorders and other respiratory conditions are taken into account. Chronic hypercapnic respiratory failure in DM1 patients occurs because of an imbalance in respiratory muscle load, pulmonary load and central respiratory drive [19]. It is important to be aware that respiratory muscle weakness can occur independently of involvement in other muscles. Furthermore, central hypoventilation with hypercapnia may develop even without pulmonary function impairment, presumably related to central CO_2_ insensitivity.

Respiratory involvement is generally slowly progressive over years, with starting of respiratory hypoventilation during nighttime, later increasing to daytime as well. Complaints like disrupted sleep, spontaneous dyspnoea at night, fatigue, excessive daytime sleepiness, early morning headaches and concentration problems may result from sleep-related breathing disorders, including sleep apnoea and (chronic) hypoventilation. An impaired cough results in sputum stasis, atelectasis and the risk of developing (aspiration) pneumonia. Progressive respiratory muscle weakness is the main reason for development of life-threatening complications like pneumonia and respiratory failure, which are the number one cause of death in DM1 [8]. 

#### Cardiac manifestations

Cardiac manifestations are among the most common systemic features of DM1 and include conduction system slowing, atrial arrhythmias, ventricular arrhythmias, cardiomyopathy, and heart failure, which are all typically gradually progressive. Although not every DM1 patient will develop cardiac complications, the incidence of cardiac abnormalities is high, potentially affecting over 50% of patients [20]. Importantly, only one fourth of patients with ECG abnormalities experiences cardiac symptoms such as recurrent dizziness, palpitations, chest pain or syncope [10]. Cardiac conduction system disturbances can have sudden and catastrophic presentations and the annual incidence of sudden death has been estimated at approximately 1% [21]. As sudden cardiac death can be a presenting symptom, this is an important question to ask regarding family history.

#### Neuropsychological and cognitive symptoms

Involvement of the brain in DM1 is almost as variable as the disease itself and includes alterations in personality, affective and cognitive disorders, and behavioural and sleep disorders [22, 23]. A highly characteristic feature is reduced initiative or apathy, along with limited awareness of the illness. This may make it challenging to support individuals in adhering to therapy and making behavioural changes. Cognitive functions can be affected in various ways, often including visuospatial and executive abilities [22]. Together, these cognitive deficits and behavioural changes often result in serious impairments in social and functional domains of living and increased caregiver burden [22, 23]. 

#### Gastrointestinal symptoms

In DM1 both smooth and striated muscles are involved, including the gastrointestinal tract. Gastrointestinal symptoms are very common in DM1. Problems with stool consistency consist of both constipation and diarrhoea, even alternating within the same patient. Other frequently reported symptoms are abdominal pain, bloating, and faecal incontinence [24]. Recurrent pseudo-obstruction is another recognised problem that may sometimes be mislabelled as ‘cyclic vomiting’. These symptoms are partly related to weakness of the pelvic floor and anal sphincter, but also the gastrointestinal nervous system may play a role. It is important to acknowledge that these symptoms can significantly impact a patient’s quality of life and may have led to prior consultations with a primary care physician or gastroenterologist.

#### Fertility issues

DM1 can affect fertility in both women and men.

In women, fertility may be reduced due to menstrual irregularities, decreased ovarian reserve, and poor response to ovarian stimulation [25]. As a result, patients may consult a reproductive medicine specialist and undergo in vitro fertilisation (IVF) or hormone therapy to achieve pregnancy. These fertility treatments sometimes occur without a known DM1 diagnosis, posing a risk of transmitting the disease to the child, often in a more severe form. Reproductive medicine specialists should therefore be aware of the signs of DM1 and consider genetic testing when encountering unexplained fertility problems, muscle symptoms, or family history suggestive of neuromuscular disease.

In men, fertility issues are common and often more severe. Many affected men are subfertile or infertile. Testicular atrophy, low testosterone levels, and primary testicular failure are frequently observed, with oligospermia or azoospermia reported in many cases [26–28]. In both sexes, pregnancy and conception require careful counselling. Pregnancy in DM1 is associated with increased maternal and fetal complications, including ectopic pregnancy, polyhydramnios, placenta praevia, preterm delivery, caesarean section, and lower Apgar scores at birth [25, 29]. Maternal muscle weakness and respiratory involvement can further complicate labour and postpartum recovery, necessitating multidisciplinary care.

#### Other

As shown in Table 1, DM1 constitutes many more symptoms than the ones described here. This includes, but is not limited to, urinary incontinence, endocrine dysfunction such as diabetes and thyroid abnormalities, and issues with oral health [30, 31]. Although not all symptoms are discussed in detail in this review, it is important for physicians to realise how broad the spectrum of symptoms possibly can be in DM1.

### Late-onset DM1

The late-onset subtype refers to patients who present with first symptoms after 40 years of age [7]. These patients generally have a milder phenotype. Frequent symptoms include (early-onset) cataracts, fatigue and mild muscular involvement [10]. Late-onset patients may not even be aware that they have the disease, especially when they are the first generation carrying DM1 in a family. A diagnosis may only come to light once a child with a more severe phenotype is born in a later generation. Early-onset cataract is the most common presenting symptom and should alert an ophthalmologist to consider further analysis.

Although the muscular symptoms are usually milder compared to the adult form of the disease and do not lead to severe limitations in activities of daily living, late-onset DM1 is not a benign condition [10]. The prevalence of cardiac conduction defects is similar to the adult subtype and clearly more prevalent than in a healthy population of the same age. Respiratory involvement occurs less frequently in the late-onset subtype, but still 17% requires non-invasive ventilation [10]. Survival is higher in the late-onset subtype compared to other types of DM1, but it is unknown if it differs from the general population [8, 32]. 

### Congenital DM1

**Fig. 2:**
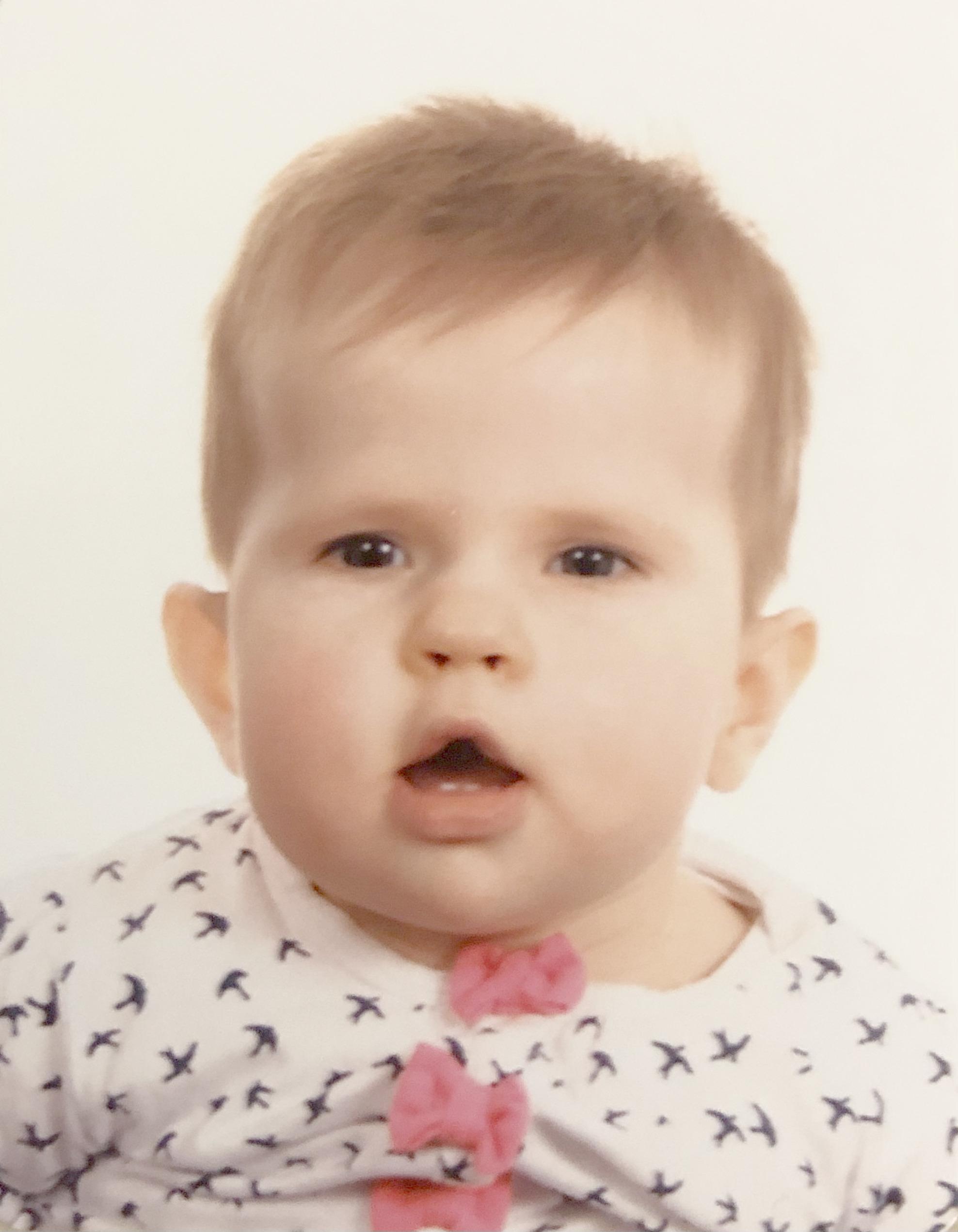
Photograph of an infant diagnosed with congenital DM1, showing the characteristic ‘tent-shaped’ mouth

Congenital DM1 is the most severe form of the disease. Characteristic symptoms that are seen after delivery include hypotonia, muscle weakness, feeding difficulties and mechanical respiratory failure [29]. Clinical features that may readily be noticed include a ‘tent-shaped’ mouth, a weak cry, or poor sucking, all of which can be signs of facial weakness (Fig. 2). Respiratory failure is a serious issue and probably results from a combination of diaphragmatic weakness, pulmonary hypoplasia, reduced central respiratory control and aspiration pneumonia [33]. In these patients, X-ray may show a raised diaphragm as a sign of respiratory muscle weakness. In many cases intubation and mechanical ventilation are required directly after birth, and can be necessary for a substantial period. Neonatal mortality is described in 16% of patients, with respiratory failure as the main cause of death [29]. Another key feature of congenital DM1 are feeding problems and swallowing disorders. It is not uncommon to require feeding therapy for several weeks, sometimes even via parenteral nutrition. Other gastrointestinal symptoms include gastroesophageal reflux, gastroparesis and obstipation. Musculoskeletal deformities can also occur in congenital DM1. The most common musculoskeletal finding is talipes equinovarus, which is present in about one third of patients [29]. Spinal deformities (such as scoliosis), joint contractures, and various types of foot deformities are less frequent but can also be observed. Thin ribs are associated with DM1, but may occur in other neuromuscular disorders as well [34]. In male patients, cryptorchidism is a common finding although not very specific for DM1. Brain ultrasound can show ventriculomegaly, as it is a commonly reported neuroradiological feature in DM1 [29]. 

Several manifestations of the disease can already be present and detected prenatally. However, if the mother is unaware that she has DM1, diagnosis of DM1 is challenging during pregnancy [29]. Polyhydramnios, caused by reduced fetal swallowing, is the most common feature and is also described in several other neuromuscular disorders. Other ultrasonographic features include facial weakness represented as a ‘tent-shaped’ mouth and congenital talipes equinovarus [35]. Especially if all three signs are present this can be suggestive of DM1, and a careful examination of the mother for subtle symptoms may be helpful in these cases. Additionally, hypotonia is a common phenomenon which is reflected by decreased fetal movements. DM1 can easily be misdiagnosed as hypoxic-ischemic encephalopathy (HIE) due to the low Apgar scores at birth. Therefore, umbilical cord blood gases and other indicators of HIE should be carefully considered when assessing the neurological presentation at birth. Children that survive the neonatal period generally improve in the years thereafter, although motor and cognitive milestones are delayed.

In later life, starting from adolescence, the motor symptoms may show increasing similarities to the adult-onset phenotype.

### Childhood DM1

Patients with childhood DM1 have clinical onset of symptoms between 1 month and 18 years of age [7]. Because many of the characteristic symptoms of DM1 such as myotonia or cataracts are often not present in early childhood, diagnosis can be especially challenging. The first presenting symptoms may be a delay of motor milestones or hypotonia on physical examination. However, motor development can also be normal and muscle strength typically remains stable until adolescence [36]. Speech and language delay and school difficulties related to cognitive impairment are very common and may often be the sole manifestation of the disease. Approximately half of children with DM1 have at least one DSM-IV psychiatric diagnosis, with a wide variety ranging from depression, anxiety, and attention deficit hyperactivity disorders to avoidant personality types and social communication disabilities [36, 37]. 

Gastrointestinal symptoms pose a large problem and may manifest as faecal incontinence, constipation or recurrent diffuse abdominal pain [36]. Retrospective evaluation of the perinatal period may help to find clues such as polyhydramnios, transitory feeding difficulties or abnormalities at birth. Physical examination should be focused on any signs of muscle involvement, including observation of gait to assess for weakness in the dorsiflexors of the feet and presence of subtle signs of facial weakness, ptosis or dysarthria. Additionally, it is important to pay attention to musculoskeletal deformities such as scoliosis, joint contractures and foot abnormalities.

Starting from the adolescent age (age 10 and above), children may begin to exhibit overlap with both the childhood DM1 and the adult-onset phenotype. Neurodevelopmental disorders and intellectual impairment often play a major role in this group. Myotonia and the myopathic facial features, which are uncommon at a younger age, are seen more often after the first decade [36]. With puberty, fertility disorders such as irregular periods, hypermenorrhoea or prolonged episodes of amenorrhoea can become apparent [25]. Visual symptoms are rare in childhood forms of DM1, but ophthalmic examination may reveal ocular findings such as hyperopia, strabismus and early signs of cataract [38]. With clinical manifestation at a later age, the phenotype increasingly resembles the adult form.

### Genetics and genetic testing

DM1 is caused by a CTG repeat expansion in the 3′ untranslated region of the *DM1 protein kinase* (*DMPK*) gene on chromosome 19q13.3. The CTG size ranges from 5 to 35 in the normal population and is increased in DM1 from 50 up to several thousands. When the abnormal CTG repeat in the DNA is transcribed into CUG repeats in the RNA, these expansions sequester RNA-binding proteins, disrupting the splicing mechanism. This leads to the production of multiple mis-spliced, non-functional proteins, which likely explains the wide range of clinical symptoms in DM1 [2]. This is different from most monogenic disorders, where symptoms generally arise from a single defective protein.

As the CTG repeat expansion in the *DMPK* gene increases, the disease tends to present more severely and at an earlier age [2]. However, there is substantial overlap in CTG repeat sizes among different subtypes. This overlap can be partially attributed to significant variations in CTG repeat length across various organs because of the somatic instability of the repeat [2, 39]. Additionally, because of its instability the repeat lengths increase over time. Finally, not only the length of the CTG repeat, but also the presence or absence of interruptions within the repeat, has an effect on the clinical phenotype [40]. Taken all of the above into account, repeat size cannot be readily used as a prognostic indicator to predict disease severity on an individual patient level.

The genetic transmission of DM1 is characterised by anticipation, where the severity of symptoms increases and the age of onset decreases with each successive generation. This is again because of the instability of the expanded CTG repeat sequence, which tends to grow larger in subsequent generations. Even individuals with only a small repeat expansion in the *DMPK* gene can experience this phenomenon. These limited expansions, known as premutations (36–50 repeats) and protomutations (51–80 repeats), may remain asymptomatic although cardiac and respiratory complications can occur in some of these patients (Table 2) [41]. However, these individuals carry the risk of passing on an expanded allele to their children, potentially leading to a more severe form of the disease. The patterns of genetic transmission differ between men and women. In the case of pre- and protomutations, male carriers are more likely to transmit a larger CTG repeat sequence [41]. However, in children born with the congenital form of the disease, which typically involves a large repeat expansion, there generally is a maternal transmission.

If DM1 is suspected, diagnosis can be confirmed by genetic analysis showing an expansion of CTG repeats in the *DMPK* gene. Testing should ideally be achieved using targeted molecular assessment of the *DMPK* gene, as repeat expansions may be missed or misinterpreted on high yield genetic sequencing such as whole exome or genome sequencing [42]. Any patient with suspected or confirmed DM1, along with their family members, should be offered a referral to a geneticist. Considering the clinical variability of the condition, it is important to review the family history carefully and have an informed discussion about testing options.

### Ancillary investigation findings

The history and physical examination followed by genetic testing currently form the cornerstone for diagnosis of DM1. When a phenotype is unclear and includes a broader differential diagnosis, other diagnostic tests may be performed earlier in the process. Some ancillary findings may add to a suspicion for diagnosis of myotonic dystrophy. Electromyography studies are generally considered most helpful and can reveal myotonic discharges. These myotonic discharges are most prominent in distal limb muscles. However, myotonic discharges on EMG do not correlate well with clinical manifestation of myotonia and can be present on EMG without any clinical signs of myotonia [43]. Laboratory studies may show several abnormalities, although none of these are specific for DM1. These include immunoglobulin G (IgG) deficiency, elevated liver enzymes, hypercholesterolaemia, hypertriglyceridaemia, hyperglycaemia and (para)thyroid abnormalities [30, 44, 45]. Serum creatine kinase can be slightly elevated in patients with DM1 but is often normal [46]. NT-proBNP may be increased in cases with cardiac involvement, and common ECG abnormalities include atrioventricular conduction disorders, right/left bundle branch blocks, prolonged QTc and supraventricular arrhythmias [20, 47]. Although muscle biopsy is generally not performed for DM1 given the availability of genetic testing, it can show abnormal histopathological changes in affected muscles including type 1 atrophy and fibrosis [48]. 

### Current management

There is currently no therapy to stop or slow down the underlying disease process of DM1. Management is focused on treatment of symptoms and prevention of complications. Given the multisystemic nature, management of DM1 requires a comprehensive team-based approach often involving a wide range of healthcare professionals, including different medical specialists, physiotherapists, occupational therapists, and psychologists. Therefore, the importance of multidisciplinary collaboration and close communication is crucial in this context. In 2018 consensus-based care recommendations for both adult and childhood DM1 were developed to guide clinical management [5, 6]. Patients with DM1 suffer from a lack of initiative and decreased disease awareness [9]. These features can keep DM1 patients from assertively requesting the care they need, coming to scheduled clinical visits or adhering to medical advice. Consequently, healthcare providers may perceive caring for DM1 patients as challenging. The management of DM1 patients requires clinicians to proactively and systematically search for signs and symptoms of involvement of various organs.

Muscle symptoms should periodically be evaluated for any significant change or disability. Severe myotonia can be treated pharmacologically with mexiletine although caution should be taken for cardiac side effects [5, 6]. Given the potential life-threatening complications, cardiac and respiratory evaluations should be conducted at least annually in all patients. These evaluations should include a 12-lead ECG and pulmonary function tests. Patients should be referred to a cardiologist experienced in DM1 for ongoing follow-up, including a 24-hour Holter monitor and echocardiography every 2 to 5 years. Respiratory function should be monitored regularly using forced vital capacity (FVC) measurements to track any progression of respiratory weakness, with referral to a pulmonologist if symptoms develop. If nocturnal hypoventilation is suspected, polysomnography with CO_2_ testing should be performed, as some patients may ultimately require nocturnal or even continuous non-invasive positive-pressure ventilation. Annual influenza vaccination is recommended. Stimulant therapy with modafinil can be considered if central hypersomnia with excessive daytime sleepiness is suspected, but generally does not treat fatigue [5, 6]. Depending on the patient’s symptoms, referral to an ophthalmologist, endocrinologist, gastroenterologist, rehabilitation specialist, fertility expert or mental healthcare professional may be indicated. Furthermore, genetic counselling should be offered to patients and their relatives. Especially for women of childbearing age or male patients in the fertile phase of life, it is important to provide parental counselling on topics such as prenatal genetic diagnosis and preimplantation genetic testing. For new patients, it is helpful to inform them about the Myotonic Dystrophy Foundation, an international organisation offering support, education, and resources for those with DM1, as well as the various national patient associations available.

An important aspect to consider is that patients with DM1 are more likely to have adverse reactions to medications used for anaesthesia and analgesia with sometimes severe consequences. Therefore, special caution should be taken in patients with DM1 who are undergoing surgery or any other intervention. Specific recommendations regarding anaesthesia can be found in the consensus-based international care recommendations [5, 6]. 

Lastly, given the chronic progressive nature of the disease and the shortened life expectancy, advanced care planning is also an important aspect of management. There has been increasing attention in recent years to advanced care planning for patients with DM1, and a guideline containing specific recommendations has recently been published [49]. 

**Table 2:** Genetic and clinical subtypes of DM1

	Age of onset	Main clinical features
Premutation	-	Asymptomatic
Protomutation	-	Mainly asymptomatic or late-onset phenotype At risk for cardiac conduction defects and respiratory involvement
Late-onset	> 40 years	Cataracts Fatigue Mild muscle symptoms Cardiac and respiratory involvement
Adult-onset	18– 40 years	See Table 1
Childhood	1 month − 18 years	Developmental delay Cognitive and behavioural impairments Gastrointestinal symptoms Dysphagia, dysarthria Fatigue Difficulty with fine motor skills Ventriculomegaly (neonates)
Congenital	< 1 month	Hypotonia Feeding difficulties Respiratory failure Musculoskeletal deformities Severe developmental delay Severe cognitive impairment

## Conclusion

Although named only after its neuromuscular symptoms, DM1 is characterised by multiorgan involvement and can present with a broad variety of symptoms. Knowledge of the (many possible) clinical findings can help clinicians fit pieces in the patient file together to come to a, sometimes puzzling, diagnosis of DM1. It is important to raise awareness of this disease among healthcare professionals as a timely diagnosis is important for adequate management and prevention of potentially severe complications.

## Data Availability

Not applicable.

## Abbreviations

ADHD: Attention deficit hyperactivity disorder
CTG: Cytosine-guanine-thymine triplet
DMPK gene: Myotonic Dystrophy Protein Kinase gene
DM1: Myotonic Dystrophy type 1
DSM-IV: Diagnostic and Statistical Manual of Mental Disorders, 4th edition
ECG: Electrocardiogram
EMG: Electromyography
FVC: Forced vital capacity
HIE: Hypoxic-Ischemic Encephalopathy
IgG: Immunoglobulin G
IVF: In vitro Fertilisation
NT-proBNP: N-terminal pro b-type natriuretic peptide
OSA: Obstructive sleep apnoea
